# Methodological Issues in Internet-Mediated Research: A Randomized Comparison of Internet Versus Mailed Questionnaires

**DOI:** 10.2196/jmir.1593

**Published:** 2011-12-12

**Authors:** Lisa Whitehead

**Affiliations:** ^1^Centre for Postgraduate Nursing StudiesUniversity of OtagoChristchurchNew Zealand

**Keywords:** Paper-and-pencil questionnaire, equivalence, questionnaire

## Abstract

**Background:**

The majority of Internet-mediated studies use measures developed as paper-and-pencil measures or face-to-face-delivered material. Previous research suggests that the equivalence between online and offline measures must be demonstrated rather than assumed.

**Objective:**

The objective of this study was to explore the equivalence 4 measures completed in an online or offline setting.

**Methods:**

A sample of students (n = 1969) was randomly assigned to complete 4 popular scales (the SF-12v2, the Hospital Anxiety and Depression Scale (HADS), the Fatigue Symptom Inventory, and a single-item fatigue measure) either online or by mail survey (pencil and paper). The response rate was 52.51% (n = 1034) and comparable between the online and offline groups.

**Results:**

Significant differences were noted in fatigue levels between the online and offline group (*P* = .01) as measured by the Fatigue Symptom Inventory, with the online sample demonstrating higher levels of fatigue. Equivalency was noted for the SF-12v2, the Hospital Anxiety and Depression Scale, and the single-item fatigue measure. Internal consistency was high except for the SF-12v2. The SF-12v2 may not be an ideal measure to use for remote administration.

**Conclusions:**

Equivalency of the Hospital Anxiety and Depression Scale (HADS) and the Physical Component Score and Mental Component Score of the SF-12v2 for online and offline data were demonstrated. Equivalency was not demonstrated for the Fatigue Symptom Inventory. Explanations for the difference in fatigue score between the online and offline samples are unclear. Research that seeks to match samples and control for extraneous online and offline variables is called for, along with exploration of factors that may mediate the completion of questionnaires or alter the respondents’ relationship with the same, to enhance progress in this area.

## Introduction

An Internet-mediated approach to conducting research in the field of health affords researchers a myriad of advantages, including the ability to reach traditionally difficult-to-access groups such as rural populations, people living with illness and disability, and shift workers, and widens geographical access [1,2]. The Internet offers another route of participation in studies for those unable to leave their homes and for those who find reading common forms of print difficult [3]. The Internet may help to defuse embarrassment, feelings of being judged, or shyness [4] and may enhance disclosure [5]. Internet studies present fewer barriers to participation such as keeping appointments or putting a questionnaire in the mail [6].

While there is evidence that online tests can be reliable and valid [7,8], there is also evidence that psychometric properties may change subtly when a test is placed on the Web [9]. The evaluation of a 5-factor personality inventory [10] found that a small number of the items loaded on different factors (ie, different factors from those they had loaded on in the offline development sample). Inflated results have been noted on the Hospital Anxiety and Depression Scale (HADS) [11] when completed online [12]. Counter to such results, a study of the equivalency of 16 scales noted no significant difference or trends in the completion of the scales [13], and a study of scales used across 16 countries found no discernible differences either [14]. Equivalence of mental health questionnaires (General Health Questionnaire, Symptom Checklist, Medical Outcomes Study Social Support Survey, Perceived Stress Scale, and Utrecht Coping List) found fair to excellent intraclass correlation coefficients (.54–.91) [14].

A key question is, that if indeed differences exist in the distribution of scores generated from online and offline measures, how critical are these? The distribution of scores becomes particularly important if clinical cut-off points are to be generated from the data collection.

The majority of studies that have been conducted in this field have drawn on existing findings, often poorly matched to the online study group and convenience samples. Few studies generate randomized samples assigned to online or offline completion. Issues of sampling bias must be taken into account when interpreting the results of many studies.

The aim of this study was to explore the equivalence of 4 self-report measures administered in an online and offline (paper version) setting.

## Methods

### Participants

A sample of 2000 students was randomly selected from a database containing all students enrolled at a university (N = 20,688) and then randomly assigned to either the online or offline completion group. This process was undertaken by a biostatistician independent of the study using the randomization feature in Excel 2007 (Microsoft Corporation, Redmond, WA, USA). Of the 2000 students selected, it was established that 31 students had left the university; the final denominator was 1969 (Table 1).

To enhance the response rate, three follow-ups were sent, unless a participant declined to participate (n = 18). The sample closely matched the wider student population by gender, ethnicity, and makeup of home and overseas students (Table 2).

**Table 1:** Participant response rates to online versus mail questionnaires

	Selected	Left the university	Final denominator	Declined participation	Unable to deliver
Online	1000	14	986	1	23
Mail	1000	17	983	17	32

**Table 2:** Characteristics of study sample (total sample, online sample, and mail sample) and total study population

Sample	Male		Female		White		Home student		Overseas student	
	n	%	n	%	n	%	n	%	n	%
Total	373	39.4%	594	61.4%	665	68.2%	842	87.3%	124	12.7%
Online	180	38.3%	290	61.7%	319	67.9%	403	85.9%	66	14%
Mail	193	38.8%	304	61.2%	346	69.9%	439	88.6%	58	11%
Study population	8509	42.50%	11,511	57.50%	13,694	68.40%	17,618	88.0%	2402	12.0%

### Procedure

The participants who were randomly assigned to participate by mail questionnaire were sent a letter of introduction and the questionnaire to their home address. A stamped, self-addressed envelope was also included, and participants were asked to complete the questionnaire and return this as soon as possible. Two further reminders were sent by mail to those who had not returned a questionnaire 2 weeks after the initial mailing, and then 2 weeks later. No further reminders were issued after this time.

The participants who were randomly assigned to participate by online questionnaire were emailed an invitation to participate. The email contained a Web link that when clicked took the participant to the questionnaire sited on the university’s intranet. The questionnaire was not accessible except through the link provided in the email. The questions were presented 6 to a page and in the same order as in the paper questionnaire. Participants were required to complete all questions and to submit each page, which then automatically brought up the next page of questions. Participants were not able to go back and view responses or change these once they had submitted the page.

### Data Collection

The questionnaire contained the HADS [11], the SF-12v2 [15,16], a single fatigue item [17], and the Fatigue Symptom Inventory (FSI) [18].

The HADS [11] is a widely used instrument designed to briefly assess anxiety and depression in nonpsychiatric populations. The HADS comprises 14 items, and 2 subscales with 7 items related to anxiety and 7 items to depression.

The SF-12v2 [15,16] is a measure of functional health across 8 domains and is used worldwide. A Physical Component Score (PCS) and Mental Component Score (MCS) can be calculated from the items.

The single fatigue item from the Zung Self-Rating Depression Scale, “I get tired for no reason,” has been used to screen for cancer-related fatigue among 52 patients attending an ambulatory oncology clinic [17]. Sensitivity of 78.95% and specificity of 87.88% were noted when the cut-off point for fatigue was set at 3 (“A good part of the time”) and above, when measured against the FSI. Limitations include the generalizability of this scale to detect fatigue in different settings and for different client groups.

The FSI [18] contains 14 items, each with a 10-point scale designed to measure the intensity and frequency of fatigue and its disruptive impact on quality of life. The FSI was developed with a group of patients with breast cancer and a comparison group of healthy people with no history of cancer. The scale has been further used in an outpatient sample of men and women with a variety of cancer diagnoses [18]. The results indicated that the scale was able to discriminate between people with cancer experiencing fatigue and healthy, disease-free controls, supporting the construct validity of the scale. In addition, the instrument was not keyed to a specific illness, although the scale requires further use to validate its applicability to a range of conditions.

### Data Analysis

Data were entered into SPSS version 17 (IBM Corporation, Somers, NY, USA). The internal consistency of each measure was explored using the Cronbach alpha coefficient, mean differences were explored using independent *t* tests, and the effect size of any significant differences were explored using the Cohen d.

### Ethical Considerations

The proposal was approved by a University of Otago ethics committee. Return of the questionnaire was taken as consent to participate. The data returned were anonymous; the researcher could not trace the student by response.

## Results

### Response Rate

Of the 2000 students randomly selected for the study, 31 had left the university. The final denominator was 1969 (Table 1). A few students (n = 18) chose not to take part in the study and informed us by return mail. A total of 55 questionnaires were undeliverable. The response rate, based on the final denominator, was 52.51% (n = 1034). The response rate was higher in the online group (n = 536, 54.4%) than in the mail group (n = 498, 50.7%); however, a review of the completion of questions across the questionnaire (Table 3) shows gradual attrition in the online group who completed the questionnaire online. This was not seen in the mail group. The single fatigue item, the last question before the demographic section, had a higher response rate in the mail group (n = 497, 50.6%) than in the email group (n = 472, 47.9%).

### Sample Characteristics

The mean age of participants was 24.07 (SD 8.5) years. The mean age was 23.57 (SD 7.63) years among participants who competed the online questionnaire and 24.54 (SD 9.24) years for the mail questionnaire. There were no significant differences between the online and mail questionnaire groups by age, gender, home or overseas student status, or ethnicity.

### Internal Consistency of the Measures

The internal consistency of the subscale of each measure was explored for each sample (Table 4). All scales, except for the SF-12v2, demonstrated good internal consistency in both the online and offline setting.

### Distribution of Scores on the Single-Item Fatigue Measure

The distribution of scores on the single-item fatigue measure (Table 5) did not differ significantly between the online and mail groups (c^2^_1_ = 0.1, *P* = .79, Cochran-Armitage test for trend).

### Mean Difference by Measure for Online and Mail Groups

The mean score on each measure for the online and mail groups was calculated (Table 4) and differences were explored. The only measure on which a statistically significant difference was noted was the FSI interference score. The mean fatigue interference score was higher for the online participants (mean 20.32, SD 14.59) than for the mail group (mean 18.04, SD 14.45; *t*_970_ = 2.45, *P* = .01). The effect size was very small (Cohen d = 0.07) [19].

**Table 3:** Participant response rates for individual items/scales

	Qu 1^a^		SF-12v2^b^		FSI^c^		HADS^d^		Single item^e^	
	n	%	n	%	n	%	n	%	n	%
Online	536	54.4%	488	49.5%	474	48.1%	472	47.9%	472	47.9%
Mail	498	50.7%	498	50.7%	498	50.7%	498	50.7%	497	50.6%

^a^ Start of the questionnaire.

^b^ The SF-12v2 measures physical and emotional health.

^c^ Fatigue Symptom Inventory.

^d^ Hospital Anxiety and Depression Scale.

^e^ “I feel tired for no reason.”

**Table 4:** Mean differences between measures

	Online questionnaire			Mail questionnaire			Difference	95% CI^a^	*t*	df	*P* value
	Mean	SD	Cronbach alpha	Mean	SD	Cronbach alpha					
PCS (SF-12v2)^b^	54.13	7.2	.93	54.62	6.7	.66	–0.49	–1.36 to 0.38	–1.11	984	.27
MCS (SF-12v2)^c^	46.04	9.67	.66	46.28	10.02	.67	–0.22	–1.45 to 1.0	–0.36	984	.72
FSI^d^ interference score	20.32	14.59	.93	18.04	14.45	.94	2.27	–0.39 to 0.54	2.45	970	.01
Anxiety	6.39	3.68	.80	6.31	3.72	.80	0.07	–0.105 to 0.66	0.31	968	.75
Depression	3.52	3.04	.76	3.24	3.05	.76	0.28	0.45 to 4.09	1.42	968	.16

^a^ Confidence interval.

^b^ Physical Component Score of the SF-12v2.

^c^ Mental Component Score of the SF-12v2.

^d^ Fatigue Symptom Inventory.

**Table 5:** Single-item fatigue measure (“I feel tired for no reason”) score

	0 (none or a little of the time)		1 (some of the time)		2 (a good part of the time)		3 (most of the time)		Total
	n	%	n	%	n	%	n	%	
Online	202	42.8%	212	44.9%	38	8%	20	4%	472
Mail	214	43.1%	212	42.7%	52	10%	19	4%	497
Total	416		424		90		39		969

## Discussion

Equivalency of the HADS and of the PCS and MCS of the SF-12v2 for online and offline data were demonstrated. The alpha scores for the SF-12v2 PCS scale in the mail group and the MCS scale in both groups were below the normal threshold of acceptability (.7) and indicate some uncertainty around the results of the online–offline comparisons. The SF-12v2 may not be an ideal measure to use for remote administration. The findings mainly supported those of earlier studies that have found no differences between the online and offline setting. Of note, no differences were found for the HADS, where inequivalence had been noted previously [12]. Possible reasons for the equivalence noted in this study (not noted in the previous study) were that participants were recruited from the same source and were randomly allocated to the online or offline group. Equivalency was not demonstrated for the FSI; however, the effect size of the difference in the mean scores on the FSI between the online and offline groups was very small. Explanations for the difference in fatigue score between the online and offline samples are myriad, although no one answer is likely to explain the situation. Computer aversion, computer anxiety, and computer self-efficacy have been proffered as influencing the completion of online questionnaires [9]. It is unlikely that any of these variables affected the completion of the fatigue questionnaire, where differences in the completion of the other measures were not affected, and where computer anxiety is known to be low and computer self-efficacy medium to high among university students [20]. Unlike previous studies reporting differences between data collected online and offline [21,22], the current study employed random sampling, and no obvious differences were observed between the two samples. The question of whether participants were influenced by social desirability in their response remains open; the online results may reflect greater openness to express symptoms, a phenomenon reported by other researchers [5,23], and chronic fatigue has been reported as viewed pejoratively by others [24]. However, given that self-reports of anxiety and depression, both known to be widely stigmatized, were invariant between the two data approaches, this explanation does not hold much weight either.

Questions remain around the ability to transfer an established measure for completion within an online environment without affecting the construct validity of the measure and the distribution of responses. The evidence to support differences between measures completed online and offline is not clear. There is evidence to suggest that the distribution of responses obtained from an online study may not be directly comparable with established norms. Research that seeks to match sample and control populations for extraneous online and offline variables is called for, along with exploration of factors that may mediate the completion of questionnaires or alter the respondents’ relationship with the same, if progress in this area is to be made.

## Abbreviations

FSI: Fatigue Symptom Inventory
HADS: Hospital Anxiety and Depression Scale
MCS: Mental Component Score
PCS: Physical Component Score
